# Unlocking the potential of microRNAs: machine learning identifies key biomarkers for myocardial infarction diagnosis

**DOI:** 10.1186/s12933-023-01957-7

**Published:** 2023-09-11

**Authors:** Mehrdad Samadishadlou, Reza Rahbarghazi, Zeynab Piryaei, Mahdad Esmaeili, Çığır Biray Avcı, Farhad Bani, Kaveh Kavousi

**Affiliations:** 1 Department of Medical Nanotechnology, Faculty of Advanced Medical Sciences, Tabriz University of Medical Sciences, Tabriz, Iran; 2 Stem Cell Research Center, Tabriz University of Medical Sciences, Tabriz, Iran; 3 Department of Applied Cell Sciences, Faculty of Advanced Medical Sciences, Tabriz University of Medical Sciences, Tabriz, Iran; 4 Laboratory of Complex Biological Systems and Bioinformatics (CBB), Department of Bioinformatics, Institute of Biochemistry and Biophysics (IBB), University of Tehran, Tehran, Iran; 5 Medical Bioengineering Department, Faculty of Advanced Medical Sciences, Tabriz University of Medical Sciences, Tabriz, Iran; 6 Medical Biology Department, School of Medicine, Ege University, İzmir, Türkiye; 7 Drug Applied Research Center, Tabriz University of Medical Sciences, Tabriz, Iran

**Keywords:** MicroRNA, Machine learning, Myocardial infarction, Bioinformatics, Biomarker

## Abstract

**Background:**

MicroRNAs (miRNAs) play a crucial role in regulating adaptive and maladaptive responses in cardiovascular diseases, making them attractive targets for potential biomarkers. However, their potential as novel biomarkers for diagnosing cardiovascular diseases requires systematic evaluation.

**Methods:**

In this study, we aimed to identify a key set of miRNA biomarkers using integrated bioinformatics and machine learning analysis. We combined and analyzed three gene expression datasets from the Gene Expression Omnibus (GEO) database, which contains peripheral blood mononuclear cell (PBMC) samples from individuals with myocardial infarction (MI), stable coronary artery disease (CAD), and healthy individuals. Additionally, we selected a set of miRNAs based on their area under the receiver operating characteristic curve (AUC-ROC) for separating the CAD and MI samples. We designed a two-layer architecture for sample classification, in which the first layer isolates healthy samples from unhealthy samples, and the second layer classifies stable CAD and MI samples. We trained different machine learning models using both biomarker sets and evaluated their performance on a test set.

**Results:**

We identified hsa-miR-21-3p, hsa-miR-186-5p, and hsa-miR-32-3p as the differentially expressed miRNAs, and a set including hsa-miR-186-5p, hsa-miR-21-3p, hsa-miR-197-5p, hsa-miR-29a-5p, and hsa-miR-296-5p as the optimum set of miRNAs selected by their AUC-ROC. Both biomarker sets could distinguish healthy from not-healthy samples with complete accuracy. The best performance for the classification of CAD and MI was achieved with an SVM model trained using the biomarker set selected by AUC-ROC, with an AUC-ROC of 0.96 and an accuracy of 0.94 on the test data.

**Conclusions:**

Our study demonstrated that miRNA signatures derived from PBMCs could serve as valuable novel biomarkers for cardiovascular diseases.

## Introduction

Cardiovascular diseases (CVDs) are the leading cause of human mortality, accounting for 32% of all global deaths. It is estimated that approximately 85% of CVD mortality is due to myocardial infarction (MI) [1]. MI is an acute coronary syndrome characterized by sudden blockage and stenosis of the coronary artery and subsequent myocardial ischemia, leading to extensive cardiomyocyte damage and necrosis [2].

Over the last 50 years, numerous attempts have been made to use biomarkers to facilitate diagnosis, assess the risk, follow-up therapy, and determine therapeutic efficacy in CVD candidates. Based on released guidelines, cardiac troponins (cTns) are used as a highly sensitive and accurate approach for detecting MI. Despite these inherent advantages, the high sensitivity of cTn-based assays has also led to more false-positive results [3], necessitating the advent and development of new modalities with pathological value. To improve the diagnostic value of existing MI biomarkers, a combination of complementary biological markers, such as microRNAs (miRNAs) and other genetic factors, has been proposed. Previous research supports the notion that miRNAs exhibit great potential as alternative biomarkers for CVD detection and follow-up [4]. It has been suggested that miRNAs possess 18-22 nucleotides and play a crucial role in the regulation of gene expression. Evidence indicates that miRNAs are involved in the pathogenesis of cardiac tissue injury [5]. Several biological processes, such as angiogenesis, cardiomyocyte growth and contractility, lipid metabolism, plaque formation, and cardiac rhythm, are regulated by miRNAs [6]. Circulating and tissue-specific miRNAs have shown promise as diagnostic and prognostic biomarkers across a range of cardiovascular diseases, including MI and other conditions such as CAD, heart failure, atrial fibrillation, cardiac hypertrophy, and fibrosis [7, 8]. The use of miRNAs as diagnostic and prognostic biomarkers in CVDs is supported by their stability and rapid release into circulation after myocardial injury [7]. In CAD, altered expression of miRNAs like miR-1, miR-133a, miR-208a/b, and miR-499, which are abundantly expressed in the heart, has been reported in patients compared to healthy controls. Additional miRNAs including miR-21, miR-208a/b, miR-133a/b, and the miR-30 family are frequently dysregulated in acute coronary syndrome (ACS) versus stable CAD [9]. Furthermore, miRNAs like miR-3113-5p, miR-223-3p, miR-499a-5p, and miR-133a-3p demonstrate potential as biomarkers to identify patients at risk of sudden cardiac death [10]. Moreover, miRNAs have shown diagnostic potential in other CVDs. For instance, miR-21 has been associated with cardiac injury and has been implicated in the pathology and recurrence of MI. Elevated levels of miR-21 have been observed in ACS patients and have been linked to cardiomyocyte apoptosis and cardiac hypertrophy. Similarly, miR-26 has been implicated in the pathology and recurrence of MI [11]. In addition to their diagnostic potential, miRNAs have also shown promise as prognostic biomarkers for adverse myocardial effects, sudden death, and risk assessment in MI and other CVDs. For example, miR-101 and miR-150 have been associated with flawed left ventricular contractility after MI, while miR-16 and miR-27a have been linked to an increased risk of adverse left ventricular remodeling [7, 9]. These miRNAs may provide valuable prognostic information and aid in risk stratification for post-MI complications.

Numerous studies have investigated the potential of miRNAs as biomarkers for MI, revealing promising findings. For instance, miR-1 has been proposed as a potential biomarker for MI [9]. This miRNA has shown increased expression levels in patients with MI, suggesting its potential diagnostic value. Additionally, other miRNAs, such as miR-19b-3p, miR-208a, miR-223-3p, miR-483-5p, and miR-499a-5p, have demonstrated promising diagnostic accuracy for MI within a short time window after the onset of symptoms [10]. A recent systematic review compared the peak time and diagnostic accuracy of miRNAs and conventional biomarkers in MI. The results revealed miR-1-3p, miR-19b-3p, miR-208a, miR-223-3p, miR-483-5p, and miR-499a-5p had superior peak times within 4 h and better accuracy versus cTn and Creatine kinase-MB, indicating their promise for early diagnosis. The strengths of miRNAs included their early peak expression, satisfactory sensitivity and specificity, and higher accuracy especially within the first few hours of symptom onset compared to conventional biomarkers [12].

It has been postulated that the function and diagnostic properties of miRNAs are beyond the myocardium in patients with CVD. Specifically, the expression of miRNAs can vary in different biofluids and cell components such as serum and peripheral blood mononuclear cells (PBMCs) [13]. PBMCs are a fraction of white blood cells, including monocytes, lymphocytes, macrophages, and other cells of the immune system [14]. Emerging data indicate that PBMCs can be used as a valid source of biomarkers for monitoring various pathological conditions. Of note, the alteration of mRNAs and miRNAs under pathological conditions provides valuable information about different kinds of disorders. PBMCs can recapitulate the conditions of target tissues, thus providing a highly sensitive and specific source of biomarkers [15]. Combined with these conditions, these cells are repositories of dysregulated genes and miRNA expression profiles in CVDs [14, 15].

In recent years, the advent and application of machine learning (ML) has been an exciting prospect for advancing scientific research. Although the concept of ML and its initial algorithms were conceived many years ago, recent improvements in computing power and access to vast amounts of data have demonstrated that ML techniques outperform classical statistical methods in various fields. Furthermore, the progress made in omics technologies has enabled the analysis of massive and intricate biological datasets, consisting of hundreds to thousands of samples, which makes it possible for ML to extract valuable biological insights and information from such data [16]. Consequently, ML provides innovative methods for merging and interpreting diverse types of omics data, leading to the identification of new biomarkers. These biomarkers can aid in precise disease prediction, patient stratification, and the development of novel therapeutic approaches [17].

In this study, we aimed to identify potential miRNA biomarkers in patients with MI by combining and analyzing three different microarray datasets from PBMCs. The integration of omics data with bioinformatics and ML techniques could be a promising tool in the discovery of new and more accurate biomarkers for monitoring MI. Additionally, this approach can deepen our understanding of the underlying mechanisms of MI and aid in the development of valid diagnostic biomarkers and patient stratification.

## Methods

### Microarray data collection

Microarray datasets were obtained from the Gene Expression Omnibus (GEO) database (https://www.ncbi.nlm.nih.gov/geo/). To obtain robust classification performance between MI, healthy control, and CAD samples, sufficiently large sample sizes for each group are required. For this purpose, the GSE59867 dataset was selected, as it contains sizable numbers of both MI and CAD samples. To provide an equally large set of healthy controls, the GSE56609 and GSE54475 datasets containing healthy samples were also included. Combining these three datasets enabled comparative analysis between MI, CAD, and healthy control groups with adequate statistical power. All samples were produced using Affymetrix Human Gene 1.0 ST Array platform (GPL6244). This platform contains 189 miRNA probes based on the annotation data from the GEO database. Only healthy, CAD, and early-stage MI samples were selected from these datasets for further analysis. Early-stage MI samples were analyzed to enable detection of miRNA biomarkers specific to the initial ischemia and infarction event, before extensive myocardial necrosis and remodeling occurs. Using samples from the early phase enhances identification of miRNA signals related to plaque rupture and MI onset versus stable CAD. Additionally, early-stage samples allow investigation of mechanisms initiating myocardial injury. The basic information for the three datasets evaluated in this study is provided in Table  1. Bioinformatics analyses including preprocessing, differential expression analysis, and functional and pathway enrichment analyses were conducted using R, ver. 4.2.0 [18], and RStudio [19]. All plots and graphics of these sections were created using the ggplot2 R package [20].

**Table 1:** Sample information on the GEO microarray dataset

Dataset	Platform	Healthy	CAD	MI	References
GSE59867	GPL6244	–	46	111	[21]
GSE56609	GPL6244	46	–	–	[22]
GSE54475	GPL6244	5	–	–	[23]

### Preprocessing

The raw data in the form of CEL files from all datasets were obtained from GEO. To prepare the data for analysis, we utilized the fRMA package [24] to facilitate preprocessing of individual microarray samples and their consistent combination. For each dataset, background correction was applied using the RMA algorithm, followed by quantile normalization based on the reference distribution. To account for probe-specific effects, batch effects were eliminated during summarization and gene expression variances were estimated accordingly. In cases where multiple probe sets matched the same gene, the mean log-fold change was retained. Consequently, fRMA can serve as a technique to remove batch effects across diverse datasets generated by identical microarray platforms [25]. To ensure the effectiveness of the batch effect removal, we employed principal component analysis (PCA) and relative log expression (RLE) plots to visualize the data before and after applying fRMA.

### Differential expression analysis

The barcode algorithm was introduced by McCall et al. [26], aimed to convert actual expression values into binary barcode values. Extensive sample collections were gathered and normalization was performed using fRMA across multiple platforms, including the Affymetrix Human Gene 1.0 ST Array (GPL6244) platform. By utilizing these normalized datasets, the distribution of the observed intensities for both the expressed and unexpressed genes was estimated. The determination of whether a gene was expressed or not was based on the following equation, where a value of 1 indicates expression and a value of 0 indicates non-expression:

1 $$\begin{aligned} \hat{x}_{ij} = {\left\{ \begin{array}{ll} 1 &{} \text {if } x_{ij} \ge \mu ^{ne} + C \times \sigma ^{ne} \\ 0 &{} \text {otherwise} \end{array}\right. } \end{aligned}$$

In the barcode algorithm, the normalized intensity of gene *i* in sample *j* is denoted as $x_{ij}$. A user-defined parameter, *C*, was introduced along with the standard deviation ($\sigma ^{ne}$) and mean ($\mu ^{ne}$) of the non-expressed distribution. Based on these values, the barcode representation of a sample was generated as a vector consisting of ones and zeros. The ones and zeros generated by the barcode algorithm refer to binary calls of whether or not a gene is estimated to be expressed (1) or not expressed (0) in each individual sample. The barcode function within the R fRMA package was employed to implement the barcode algorithm, utilizing the default value of *C*.

To assess the differences in expressed ratios between the MI and healthy control groups, Fisher’s exact test was performed on the barcode values of individual genes. Genes that exhibited a false discovery rate (FDR) below 0.05, calculated using the Benjamini-Hochberg procedure to account for multiple testing issues were identified as differentially expressed genes (DEGs). The same procedures were applied to the CAD versus healthy control comparison, as well as to the MI versus CAD group, to identify DEGs specific to each comparison.

#### Differentially expressed miRNAs

The differentially expressed miRNAs were defined as those miRNAs within the total DEGs (i.e. they had an FDR $< 0.05$ resulted from the Fisher’s exact test comparing the sample groups).

### Functional and pathway enrichment analyses

The R clusterProfiler package [27] was utilized to perform Kyoto Encyclopedia of Genes and Genomes (KEGG) pathway enrichment analysis and Gene Ontology (GO) functional annotation on the set of DEGs. GO analysis encompassed three categories: biological process (BP), cellular component (CC), and molecular function (MF). For statistical significance, an adjusted p-value threshold of less than 0.05 was employed. Enrichment analyses were conducted separately for DEGs specific to the MI-healthy and CAD-healthy comparisons. All the default parameters provided by the package were used in the analyses.

### ML procedure

ML analysis was performed using Python software, ver. 3.9, Numpy [28], Pandas [29], and Scikit-Learn packages [30]. Whenever hypertuning was needed, the Scikit-opt package [31] was used. In all ML analyses, the datasets were divided into training and test sets at a 0.7:0.3 ratio, and all reported results are the average of 10-fold cross-validation.

Two different approaches were used to select miRNAs for model training. The first approach was to use differentially expressed miRNAs. To capture additional miRNAs with high discriminatory power for distinguishing MI from CAD despite not reaching differentially expression criteria, a secondary approach was used. miRNAs were selected based on having individual area under the receiver operating characteristic curves (AUC-ROCs) exceeding 0.8 for separating MI and CAD. This AUC-based approach identifies miRNAs with the best classification performance, unconstrained by statistical cutoffs. Using both the differentially expressed and AUC-based selection provides complementary methods to uncover miRNA biomarkers from both a biological and diagnostic perspective.

#### Differentially expressed miRNAs

In this approach, a two-layer architecture is deployed to the data to maximize the prediction values. The first layer predicted whether a sample was healthy or not, and the second layer separated MI from CAD in the samples that were predicted as not healthy in the first layer. To this end, a distinct ML model was trained for each layer. Because there were a limited number of miRNAs in the DEGs, both layers were trained with all of them. For further comparison with the models’ performance, the ROC curve of each miRNA for classifying healthy and not-healthy, as well as CAD and MI, was generated using a logistic regression model.

##### First layer for the isolation of healthy and not-healthy samples

A support vector machine (SVM) model using RBF kernels was trained and hypertuned using all miRNAs in the DEGs. To account for the substantial class imbalance between the healthy and not-healthy groups, with 51 samples in the minority healthy class compared to 157 combined CAD and MI samples, adjustments were made to the sample weights used during model training. Without compensating for the imbalance, the machine learning model would be biased towards the majority class and potentially ignore the minority class. To counteract this, the sample weights were empirically tuned, with the weight for healthy samples set to 1 and the weight for not-healthy samples set to 0.5. These values were determined through iterative testing to produce a model with strong performance on both classes despite the imbalance. The ROC curve and confusion matrix for the model are reported.

##### Second layer for separating the MI and CAD samples

Different models were investigated to achieve the highest classification performance. To do so, SVM (with linear, polynomial, and RBF kernels), logistic regression (LR), random forests (RF), k-nearest neighbor (kNN), gradient boosting (GB), XGBoost (XGB) and decision tree (DT) models were trained. All models were trained with their preset parameters using 10-fold cross-validation. The criteria for selecting the best model were the highest accuracy and AUC-ROC for the test set. The best model was hypertuned using the scikit-opt package [31] for the best classification performance. The ROC curve and confusion matrix for the best model are reported.

#### miRNAs with the highest AUC-ROC

As in the previous approach, a two-layer strategy was employed. The first layer classified samples into healthy and not-healthy, and the second layer separated the MI and CAD samples. However, to keep the number of miRNAs as low as possible, miRNAs were selected from the second layer and their performance was evaluated in the first layer. The AUC-ROC of all miRNAs for classifying MI and CAD samples was calculated, and miRNAs with AUC-ROC $> 0.8$ were selected. ROC curves for each selected miRNA for separating healthy samples from not-healthy samples and MI from CAD samples were also plotted for further comparison.

##### First layer for the isolation of healthy and not-healthy samples

An SVM model with an RBF kernel is trained using the selected set of miRNAs. Additionally, the model was hypertuned to find the hyperparameters for the highest AUC-ROC and accuracy. The same sample weights as in the previous approach (1 for healthy and 0.5 for not-healthy samples) were used. The ROC curve and confusion matrix for the model were reported.

##### Second layer for separating the MI and CAD samples

The selected miRNA set was used to train different algorithms to determine the best model. Similar to the previous approach, the SVM (with linear, polynomial, and RBF kernels), LR, RF, kNN, GB, XGB, and DT models were trained. All models were trained with their preset parameters using 10-fold cross-validation. The models with the highest AUC-ROC and accuracy on the test set were selected and hypertuned using the scikit-opt package [31]. The ROC curve and confusion matrix for the best model were reported.

## Results

### Preprocessing

**Fig. 1:**
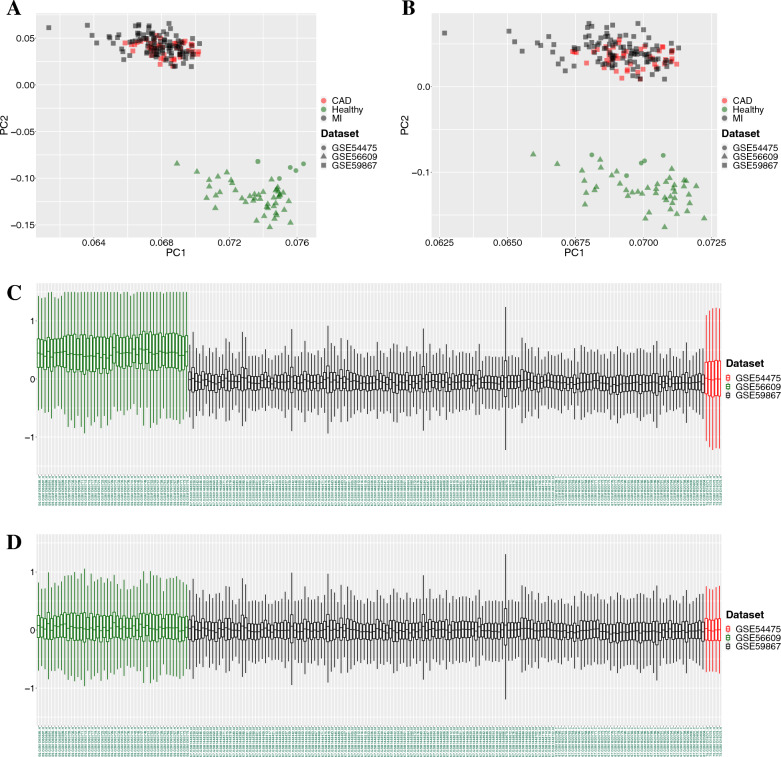
Principal component analysis plots for **A** primary data and **B** the data after fRMA, and the relative log expression plots for** C** primary data and** D** the data after fRMA

The PCA plots of the samples are shown in Fig.  1A, B. Healthy samples were separated from the CAD or MI samples in the primary data and after conducting fRMA. In the RLE plot, there was a distinct difference between the dataset means for all samples before fRMA was performed (Fig. 1C). All datasets were rearranged to approximately 0 in the RLE plot after fRMA was conducted (Fig.  1D). Moreover, there was an apparent change in the interquantile distances, but the values were still greater than 0.1.

### Differential expression analysis

**Table 2:** Total, up-, and down-regulated DEGs and differentially expressed miRNAs

	Total DEGs	Up-regulated DEGs	Down-regulated DEGs	miRNAs
MI vs. Healthy	860	323	537	hsa-miR-186-5p, hsa-miR-21-3p, hsa-miR-32-3p
CAD vs. Healthy	670	262	408	hsa-miR-186-5p, hsa-miR-21-3p, hsa-miR-32-3p
MI vs. CAD	260	144	116	hsa-miR-186-5p

According to the cutoff criterion of $FDR < 0.05$, there were 860 DEGs between MI and healthy samples. Among them, 323 were up-regulated, and 537 were down-regulated in the MI group compared to the healthy group. In the CAD and healthy group comparison, we found 670 DEGs, of which 262 and 408 DEGs were up- and down-regulated, respectively, in CAD samples. In the MI and CAD groups, the number of DEGs was 260, and the numbers of up- and down-regulated genes in MI samples were 144 and 116, respectively, compared to CAD samples. The data are summarized in Table  2.

**Fig. 2:**
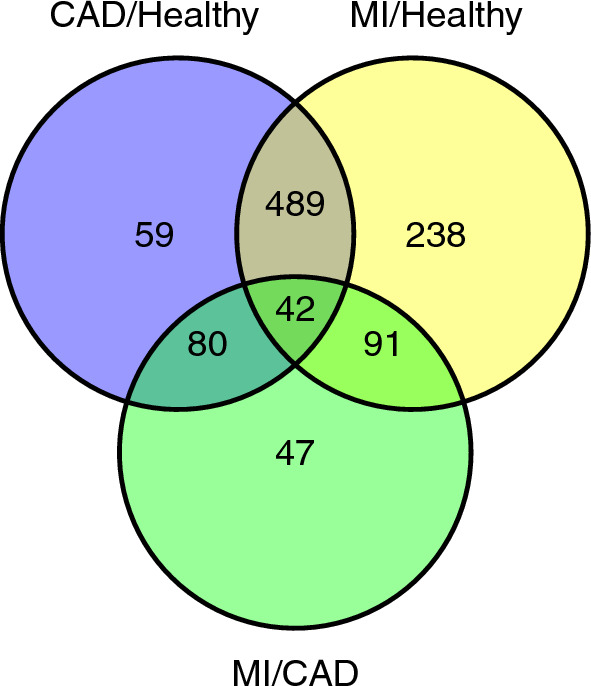
Venn diagram for DEGs in CAD/Healthy, MI/Healthy, and MI/CAD

The Venn diagram in Fig. 2 shows that the CAD and MI samples shared most of their DEGs. From 860 DEGs of MI/healthy and 670 DEGs of CAD/healthy, 531 genes were common, which is 62% of MI/healthy DEGs and 79% of CAD/healthy DEGs.

#### Differentially expressed miRNAs

Among the DEGs for MI/healthy and CAD/healthy comparison, hsa-miR-186-5p, hsa-miR-32-3p, and hsa-miR-21-3p were identified as differentially expressed miRNAs. The only differentially expressed miRNA in MI/CAD comparison was hsa-miR-186-5p (Table 2). The expression profiles of the three miRNAs are shown in Fig. 5.

### GO and KEGG enrichment analyses of the DEGs

**Fig. 3:**
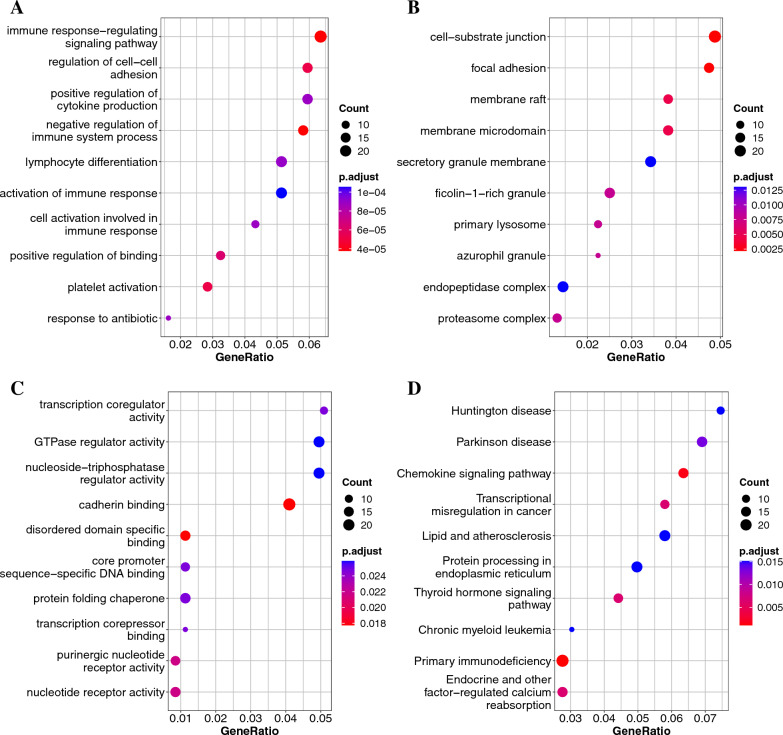
Gene Ontology (GO) and Kyoto Encyclopedia of Genes and Genomes (KEGG) pathways enriched with the MI and healthy DEGs.** A** Biological process terms.** B** Cellular component terms.** C** Molecular function terms.** D** KEGG analysis

To explore the biological classification of the DEGs, we performed GO and KEGG pathway enrichment analyses on the MI/healthy and CAD/healthy DEGs. For MI/healthy, GO enrichment analysis in the BP category suggested that the DEGs were enriched in “immune response-regulating signaling pathway,” “lymphocyte differentiation,” “immune response-regulating cell surface receptor signaling pathway,” and “leukocyte activation involved in immune response” (Fig.  3A). In the CC category, DEGs were enriched in “secretory granule membrane,” “azurophil granule,” “ficolin-1-rich granule,” “tertiary granule,” and “ficolin-1-rich granule membrane” (Fig. 3B). In the MF category, DEGs were involved in “cadherin binding” and “MHC class I protein binding” (Fig.  3C). KEGG pathway analysis indicated that the DEGs were related to the following pathways: “Chemokine signaling pathway,” “Lipid and atherosclerosis,” and “Hematopoietic cell lineage” (Fig. 3D).

**Fig. 4:**
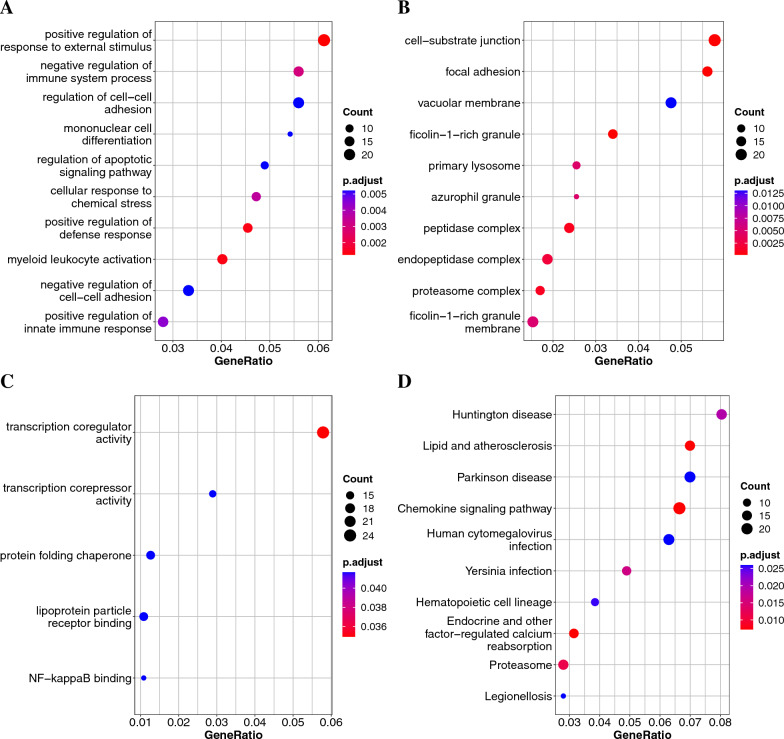
Gene Ontology (GO) and Kyoto Encyclopedia of Genes and Genomes (KEGG) pathways enriched with the CAD and healthy DEGs.** A** Biological process terms.** B** Cellular component terms.** C** Molecular function terms.** D** KEGG analysis

The enrichment results for the CAD/healthy DEGs were as follows. In the BP category, GO enrichment suggested that the DEGs were enriched in “positive regulation of defense response,” “positive regulation of innate immune response,” “mononuclear cell differentiation,” and “positive regulation of response to external stimulus” (Fig.  4A). In the CC category, DEGs were enriched in “azurophil granule,” “ficolin-1-rich granule,” and “ficolin-1-rich granule membrane” (Fig. 4B). In the MF category, DEGs were involved in “lipoprotein particle receptor binding” and “NF-$\kappa$B binding” (Fig. 4C). KEGG pathway analysis showed that the DEGs were related to the following pathways: “Chemokine signaling pathway,” “Lipid and atherosclerosis,” and “Hematopoietic cell lineage” (Fig. 4D).

### Machine learning

#### Differentially expressed miRNAs

The ROC curves of each miRNA in each layer are presented in Fig. 6. Using the logistic regression model, the AUC-ROC values of hsa-miR-21-3p, hsa-miR-32-3p, and hsa-miR-186-5p for separating healthy and not-healthy samples were 0.98, 0.99, and 0.90, respectively (Fig. 6A). The accuracy of each miRNA for classifying the samples into healthy and not-healthy groups on the test set for hsa-miR-21-3p, hsa-miR-32-3p, and hsa-miR-186-5p was 0.92, 0.98, and 0.89, respectively. The ROC curve of each miRNA for classifying MI and CAD samples is presented in Fig. 6B. The AUC-ROC and accuracy for hsa-miR-21-3p, hsa-miR-32-3p, and hsa-miR-186-5p in the test set were 0.85; 0.70; and 0.86, and 0.78; 0.67; and 0.74, respectively.

**Table 3:** Investigated miRNAs log fold-change and adjusted p-values for CAD samples relative to healthy, MI samples relative to healthy, and MI samples relative to CAD

	CAD/Healthy		MI/Healthy		MI/CAD	
	logFC	adj. p-value	logFC	adj. p-value	logFC	adj. p-value
hsa-miR-186-5p	1.4	3.60e-25	0.9	6.76e-20	−0.5	1.05e-09
hsa-miR-21-3p	1.4	1.31e-17	2.3	2.07e-47	0.8	2.96e-11
hsa-miR-32-3p	2.5	8.39e-43	2.2	3.10e-59	−0.3	7.60e-04
hsa-miR-197-5p	0.5	2.95e-20	0.7	1.59e-47	0.2	8.58e-09
hsa-miR-29a-5p	0.7	7.76e-29	0.1	1.70e-01	−0.5	2.14e-10
hsa-miR-296-5p	−0.1	5.00e-02	0.1	2.00e-02	0.2	6.15e-06

**Fig. 5:**
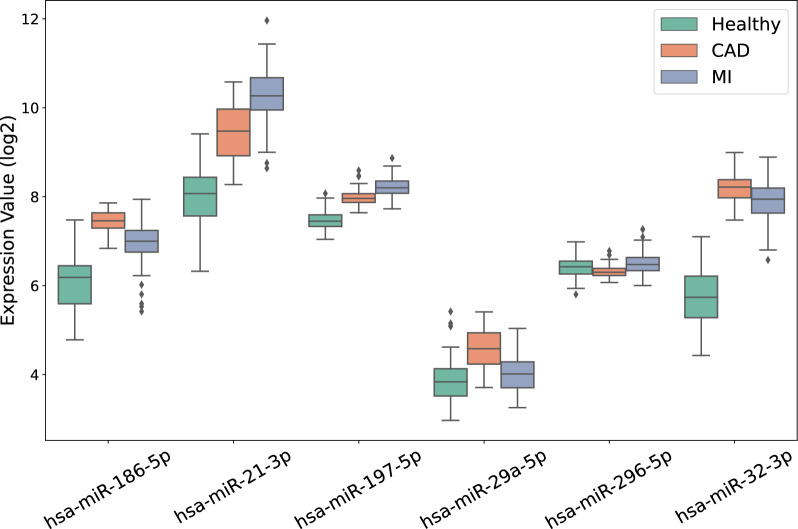
Expression profile of all miRNAs in two approaches in different sample classes

**Fig. 6:**
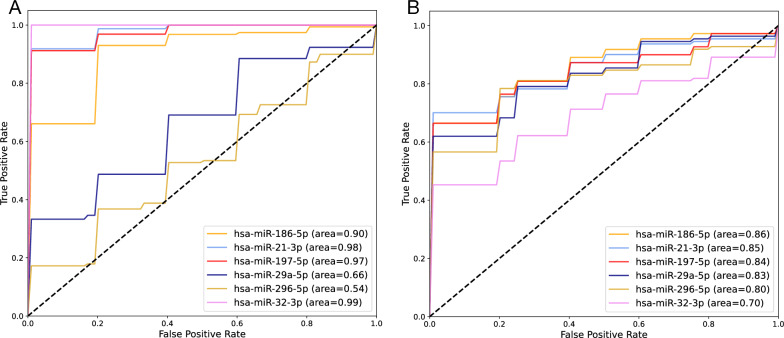
ROC curve for single miRNAs on test set classification for** A** healthy and not-healthy samples and** B** CAD and MI samples

##### First layer for the isolation of healthy and not-healthy samples

**Fig. 7:**
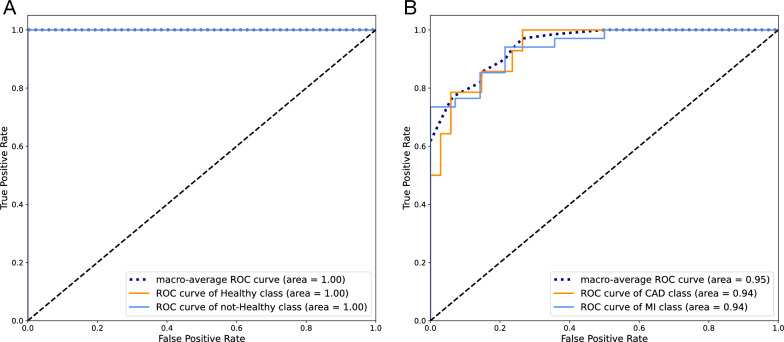
ROC curve for the model trained with differentially expressed miRNAs on test set classification;** A** An SVM model with RBF kernel for healthy and not-healthy and** B** An SVM model with linear kernel for CAD and MI sample classification

**Fig. 8:**
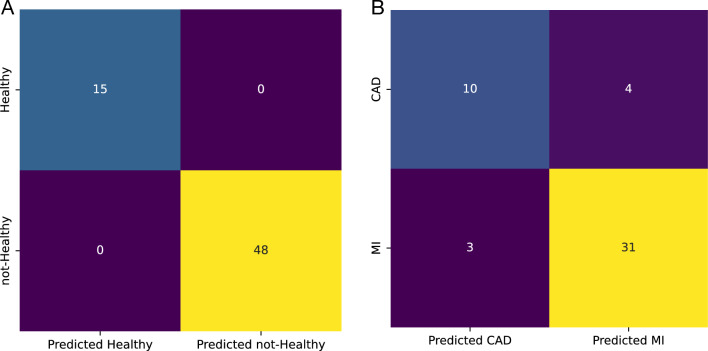
Confusion matrix for the model trained with differentially expressed miRNAs on test set classification;** A** An SVM model with RBF kernel for healthy and not-healthy and** B** An SVM model with linear kernel for CAD and MI sample classification

Although single miRNAs had an acceptable performance for this layer, their predictive value could be further improved by using them as a set. The ROC curve for the SVM model with an RBF kernel trained with all three miRNAs is presented in Fig. 7A. The model had a better performance in classification than single miRNAs.The AUC-ROC for the model was 1, and its accuracy on the test set was also 1. In Fig.  8A, the confusion matrix for the model is presented.

##### Second layer for separating the MI and CAD samples

**Fig. 9:**
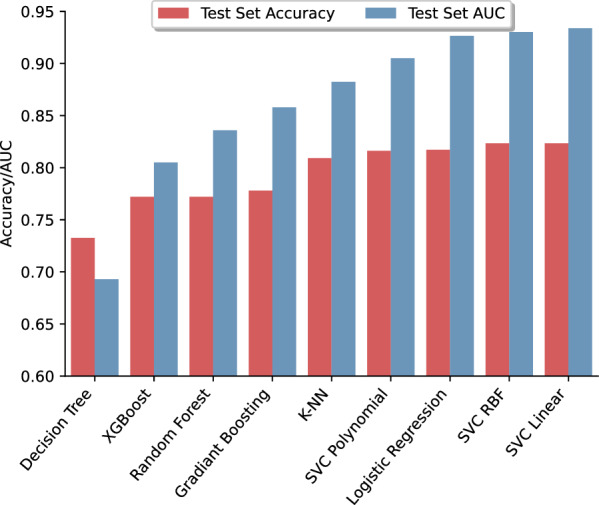
Area under the receiver operating characteristic curve and accuracy of different models trained with three differentially expressed miRNAs

Different models were trained using the expression values of three differentially expressed miRNAs. The models’ AUC-ROC and the accuracy of the test set are shown in Fig. 9. The best model from both the AUC-ROC and accuracy points of view was the SVM model with a linear kernel. The AUC-ROC and accuracy for this model with its preset values were 0.93 and 0.82, respectively. The model was hypertuned for C and gamma hyperparameters, and therefore the model showed better performance. The ROC curve of the hypertuned model is presented in Fig. 7B. For this model, the AUC-ROC reached 0.95, and the accuracy was improved to 0.85 (Table 4). Moreover, the sensitivity and specificity for the model on the test set were 0.91 and 0.71, respectively. The confusion matrix for the hypertuned model is illustrated in Fig. 8B.

**Table 4:** AUC-ROC and accuracy for SVM with a linear kernel as the best model trained with differentially expressed miRNAs on the training and test sets before and after hypertuning

Model	Metrics	Preset parameters		Hypertuned	
		Train	Test	Train	Test
SVM-linear	AUC-ROC	0.91	0.93	0.92	0.95
	Accuracy	0.83	0.82	0.84	0.85

#### AUC-ROC approach

After calculating the AUC-ROC for each miRNA to classify of MI and CAD samples, the miRNAs with AUC-ROC $> 0.8$ were selected. The miRNAs selected were hsa-miR-29a-5p, hsa-miR-197-5p, hsa-miR-186-5p, hsa-miR-21-3p, and hsa-miR-296-5p. The expression levels of these miRNAs in healthy, CAD, and MI samples are presented in Fig. 5. The ROC curves of the selected miRNAs in both layers are shown in Fig. 6.

##### First layer for the isolation of healthy and not-healthy samples

**Fig. 10:**
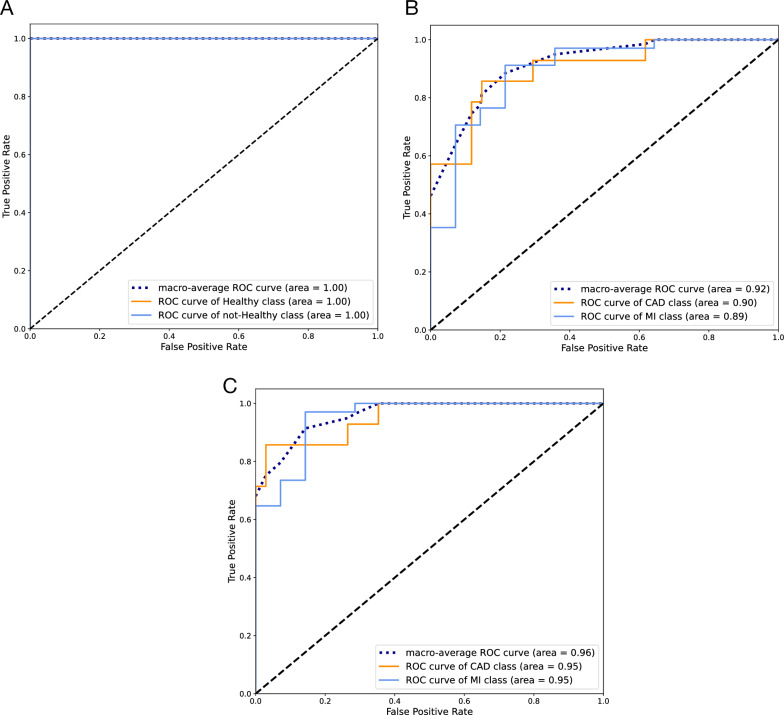
ROC curve for models trained with the set of miRNAs selected by AUC-ROC on test set classification;** A** SVM with RBF kernel for healthy and not-healthy samples classification.** B** SVM with linear kernel for CAD and MI sample classification.** C** SVM with RBF kernel for CAD and MI sample classification

**Fig. 11:**
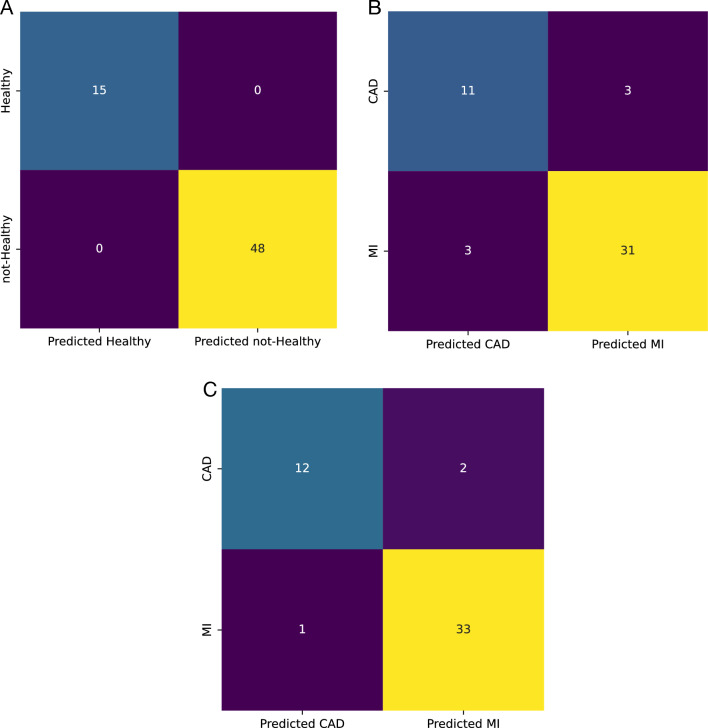
Confusion matrix for models trained with the set of miRNAs selected by AUC-ROC on test set classification;** A** SVM with RBF kernel for healthy and not-healthy samples classification.** B** SVM with linear kernel for CAD and MI sample classification.** C** SVM with RBF kernel for CAD and MI sample classification

Using the selected set, an SVM model with an RBF kernel was trained to separate healthy and not-healthy samples. The ROC curve for the model is presented in Fig. 10A, and the confusion matrix is illustrated in Fig. 11A. Both the AUC-ROC and accuracy of the model on the test set were 1.

##### Second layer for separating the MI and CAD samples

**Fig. 12:**
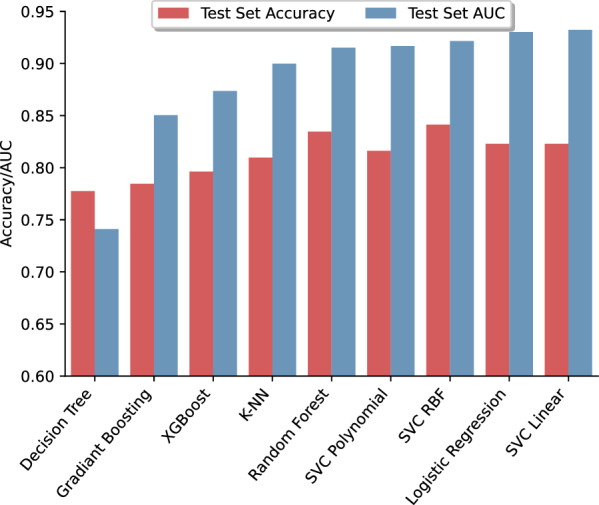
Area under the receiver operating characteristic curve and accuracy of different models trained with AUC-selected miRNAs

To find the best model for this set of miRNAs, different models were trained using their preset values. The AUC-ROC and accuracy results for the test set are presented in Fig. 12. The best model from the AUC-ROC point of view was the SVM with a linear kernel, and from the accuracy point of view, it was the SVM model with an RBF kernel. For the SVM-linear model, the AUC-ROC and accuracy were 0.93 and 0.82, respectively; and for the SVM-RBF, the values were 0.92 and 0.84, respectively. Both models were hyper-tuned, and the ROC curve for their best performance is presented in Fig. 10B, C. The AUC-ROC and accuracy for the SVM-linear model were modified to 0.92 and 0.88, respectively. For the SVM-RBF, these values increased to 0.96 and 0.94, respectively (Table 5). The sensitivities for the SVM-linear and SVM-RBF models were 0.91 and 0.97, respectively; and their specificities were 0.79 and 0.86, respectively. The confusion matrix for both models is illustrated in Fig. 11B and C.

**Table 5:** AUC-ROC and accuracy for SVM with the linear and RBF kernels as the best models trained with miRNAs selected based on their AUC-ROC on the train and test sets before and after hypertuning

Model	Metrics	Preset parameters		Hypertuned	
		Train	Test	Train	Test
SVM-linear	AUC-ROC	0.91	0.93	0.93	0.92
	Accuracy	0.85	0.82	0.90	0.88
SVM-RBF	AUC-ROC	0.90	0.92	0.96	0.96
	Accuracy	0.86	0.84	0.96	0.94

## Discussion

The prevalence of MI can lead to high mortality rates in the clinical setting. However, early diagnosis and the application of suitable treatment protocols can reduce mortality and improve MI prognosis ([1, 3, 32]). Studies have suggested that changes in miRNA expression may play a significant role in the progression of MI and the subsequent remodeling [33]. It is believed that miRNA expression is altered during the various biological processes correlated with MI within the myocardium or other related tissues [34]. Although several studies have focused on examining free circulating miRNAs in serum samples for the detection of cardiac tissue injuries [7], more information is needed to fully comprehend the miRNAs found in different blood subcomponents, such as plasma, platelets, and PBMCs. Based on previous findings, PBMCs play a crucial role in the destabilization and rupture of plaques as well as in the initial inflammatory reactions in individuals experiencing myocardial infarction (MI) [15, 35]. Moreover, PBMCs have specific miRNA profiles that are altered under certain pathological conditions, making them great candidates as disease biomarkers [15].

PBMCs can respond to several insulting conditions, such as MI, in the shortest possible time with notable changes in their miRNA profile [15]. Considering their regulatory roles, subtle changes in the transcription of miRNAs can be monitored even before alterations in mRNA and protein levels [4]. These features make miRNAs a valid early-stage diagnostic tool for the detection of minor and major cell injuries. To date, few studies have compared the miRNA profiles in PBMCs from patients with MI and other CADs and healthy samples to find a robust set of identical miRNAs to differentiate these pathological conditions.

In this study, we combined three GEO datasets for healthy, CAD, and MI samples. Having these sample sets alongside bioinformatics analysis and ML methods enabled us to identify potential biomarker sets and effective therapeutic targets. The results of the DEG analysis (Table  2 and Fig. 2) prove the close relationship between the MI and CAD samples. Interestingly, functional enrichment analysis demonstrated that DEGs in both CAD/healthy and MI/healthy were strongly correlated with the immune cell response, which is a major part of PBMCs. Two sets of miRNAs were selected as biomarker sets for sample classification. Hsa-miR-21-3p; hsa-miR-32–3p; and hsa-miR-186–5p were selected as differentially expressed miRNAs, and hsa-miR-186–5p; hsa-miR-21–3p; hsa-miR-29a-5p; hsa-miR-197–5p; and hsa-miR-296–5p were selected based on their AUC-ROC values. As shown in Fig. 6, all miRNAs selected with both approaches had AUC-ROCs $> 0.9$ for isolating healthy and not-healthy samples except for hsa-miR-296–5p and hsa-miR-29a-5p. The data confirmed that the real challenge was to classify CAD and MI samples because of the close overlap. Of the six miRNAs under investigation in both approaches, except for hsa-miR-32–3p, all miRNAs had an AUC-ROC $> 0.8$ for the discrimination of CAD and MI samples. As expected, the high AUC-ROC values of the miRNAs confirmed their high potential as biomarkers.

ML models trained with miRNA sets selected by both DEG and AUC-ROC approaches, showed better classification performance than each miRNA. To avoid unwanted complexity and poor predictive values, a two-layer architecture was designed. The first layer was used to discriminate between healthy and not-healthy samples, and the second layer was was used to separate CAD from MI candidates. As expected, in both approaches, a hypertuned SVM model could flawlessly separate healthy and not-healthy samples using distinct miRNA sets. ML models are also capable of effectively separating CAD from MI patients. Although both miRNA sets had nearly the same AUC-ROC using the best model, their accuracy, sensitivity, and specificity were different. The model trained with AUC-selected miRNAs showed better performance in all predictive values, which is logical because of the higher number of miRNAs in the set.

Numerous studies have reported that different biological processes can affect the miRNA expression in PBMCs. However, the exact role of miRNAs in the function of immune cells and the correlation between specific pathological conditions and miRNA profiles remain controversial. Several studies have proven the activation of particular miRNA types in PBMCs under cardiovascular events [36]. For instance, there is evidence that elevation of hsa-miR-186–5p suppresses the expression of cystathionine-$\gamma$-lyase, leading to the subsequent secretion of pro-inflammatory cytokines and cellular lipid accumulation. In addition, macrophage-derived hsa-miR-186–5p may promote atherosclerotic plaque formation [37]. In line with this claim, we found that hsa-miR-186–5p was up-regulated in both CAD and MI candidates compared to their control counterparts. Surprisingly, the obtained data indicated that the expression of hsa-miR-186–5p was higher in patients with CAD than in patients with MI (Fig. 5). Specifically, hsa-miR-186–5p was the only differentially expressed miRNA between CAD and MI, with a clear up-regulation in CAD, indicating its main role in the promotion of atherosclerosis.

As mentioned before, hsa-miR-21–3p was also up-regulated in both MI and CAD patients compared to healthy controls. Moreover, the expression value of hsa-miR-21–3p was significantly higher in the MI group than in the CAD group (Table 3). It is thought that the up-regulation of hsa-miR-21–3p in PBMCs is a compensatory reaction to reduce the T$_{reg}$ lymphocyte number in response to the reduction in TGF$\beta 1$ secretion into the plasma through a TGF$\beta 1$/smad-independent pathway. In line with the previous and present data, hsa-miR-21–3p can modulate the activity of PBMCs following the occurrence of cardiovascular diseases [38].

Recent data have supported the elevation of hsa-miR-32–3p levels in CAD samples with calcification of the coronary artery. Notably, hsa-miR-32–3p promotes vascular smooth muscle calcification in mice by controlling the activity of several proteins, including bone morphogenetic protein-1, runt-related transcription factor-2 (RUNX2), osteopontin, and bone-specific phosphoprotein matrix GLA protein [39]. Likewise, some reports are associated with the activity of hsa-miR-32–3p in PBMCs in several pathologies [40, 41]. The exact role of hsa-miR-32–3p in PBMCs after cardiovascular events remains unclear.

Molecular analyses have indicated the regulatory role of miRNAs selected using the AUC-ROC approach in PBMCs after a cardiovascular event. The biological importance of two common miRNAs in the DEGs and AUC-ROC approaches, hsa-miR-21–3p and hsa-miR-186–5p, have already been discussed. Based on numerous reports, hsa-miR-29a-5p can be activated in different diseases [42]. Data analysis indicated that hsa-miR-29a-5p was significantly up-regulated in CAD patients compared to the healthy and MI groups (Table 3). Increased hsa-miR-29a-5p is associated with the progression of atherosclerosis, and the combination of hsa-miR-29a-5p and ox-LDL has been suggested as a valid biomarker set for paraclinical classification [43]. However, the role of hsa-miR-29a-5p in the function of PBMCs from patients with CAD has not been thoroughly examined.

The data indicated that hsa-miR-197–5p was significantly up-regulated in both the CAD/healthy and MI/healthy groups. Previous studies have demonstrated that hsa-miR-197–5p may play a crucial role in controlling the anti-inflammatory response of IL-35 by influencing the secretion of cytokines that can either promote or suppress inflammation, the ratio of M1/M2 macrophages, and the proliferation of T$_{reg}$ lymphocytes, which are responsible for suppressing immune responses [44]. Alongside our findings, it can be concluded that hsa-miR-197–5p could be a useful diagnostic tool for predicting adverse cardiovascular events.

The findings of this study demonstrate the potential of hsa-miR-296–5p as a biomarker with high discriminatory power to distinguish between samples from individuals with MI and CAD. Hsa-miR-296–5p has been identified as a key regulator in the development and advancement of atherosclerosis by controlling the expression of target genes associated with various biological processes, including angiogenesis, cholesterol metabolism, inflammation, cellular proliferation, hypertension, and apoptosis [36]. In a previous study, hsa-miR-296–5p expression levels were found to be significantly increased in the PBMCs of CAD patients compared to healthy controls, suggesting its involvement in regulating proinflammatory cytokines such as IL-6 and TNF-$\alpha$ [45]. These findings suggested that hsa-miR-296–5p may have a significant impact on the pathogenesis of atherosclerosis and could potentially serve as a diagnostic biomarker for CAD or MI.

## Conclusion

In summary, we derived a set of miRNA biomarkers by comparing MI samples with both healthy and CAD samples. We found that the SVM model performed best in both the first layer, which separated healthy and unhealthy samples, and the second layer, which classified the MI/CAD samples. The set of miRNAs selected based on their AUC-ROC values performed better in the second layer. Overall, our two-layer structure achieved an accuracy of 0.96. This demonstrates the potential of combining bioinformatics and machine learning techniques to identify novel biomarkers and gain a deeper understanding of myocardial infarction.

## Data Availability

The datasets generated and/or analysed during the current study are available in the Gene Expression Omnibus (GEO, https://www.ncbi.nlm.nih.gov/geo/), reference numbers GSE59867, GSE56609 and GSE54475. All data generated or analysed during this study are included in this published article.

## Abbreviations

CVD: Cardiovascular disease
MI: Myocardial infarction
cTn: Cardiac troponin
miRNA: MicroRNA
ACS: Acute coronary syndrome
PBMC: Peripheral blood mononuclear cell
ML: Machine learning
GEO: Gene expression omnibus
PCA: Principal component analysis
RLE: Relative log expression
FDR: False discovery rate
DEG: Differentially expressed gene
KEGG: Kyoto encyclopedia of genes and genomes
GO: Gene ontology
BP: Biological process
CC: Cellular component
MF: Molecular function
SVM: Support vector machine
LR: Logistic regression
RF: Random forests
kNN: K-nearest neighbor
GB: Gradient boosting
XGB: XGBoost
DT: Decision tree
AUC-ROC: Area under the receiver operating characteristic curve
